# Phosphoinositide-Dependent Protein Kinases Regulate Cell Cycle Progression Through the SAD Kinase Cdr2 in Fission Yeast

**DOI:** 10.3389/fmicb.2021.807148

**Published:** 2022-01-10

**Authors:** Kun Liu, Qiannan Liu, Yanli Sun, Jinwei Fan, Yu Zhang, Norihiro Sakamoto, Takayoshi Kuno, Yue Fang

**Affiliations:** ^1^Department of Microbial and Biochemical Pharmacy, School of Pharmacy, China Medical University, Shenyang, China; ^2^Division of Food and Drug Evaluation Science, Kobe University Graduate School of Medicine, Kobe, Japan

**Keywords:** *Schizosaccharomyces pombe*, Ksg1, Ppk21, Cdr2, Cdc25, cell cycle regulation

## Abstract

Aberration in the control of cell cycle contributes to the development and progression of many diseases including cancers. Ksg1 is a *Schizosaccharomyces pombe* fission yeast homolog of mammalian phosphoinositide-dependent protein kinase 1 (PDK1) which is regarded as a signaling hub for human tumorigenesis. A previous study reported that Ksg1 plays an important role in cell cycle progression, however, the underlying mechanism remains elusive. Our genomic library screen for novel elements involved in Ksg1 function identified two serine/threonine kinases, namely SAD family kinase Cdr2 and another PDK1 homolog Ppk21, as multicopy suppressors of the thermosensitive phenotype of *ksg1-208* mutant. We found that overexpression of Ppk21 or Cdr2 recovered the defective cell cycle transition of *ksg1-208* mutant. In addition, *ksg1-208* Δ*ppk21* cells showed more marked defects in cell cycle transition than each single mutant. Moreover, overexpression of Ppk21 failed to recover the thermosensitive phenotype of the *ksg1-208* mutant when Cdr2 was lacking. Notably, the *ksg1-208* mutation resulted in abnormal subcellular localization and decreased abundance of Cdr2, and Ppk21 deletion exacerbated the decreased abundance of Cdr2 in the *ksg1-208* mutant. Intriguingly, expression of a mitotic inducer Cdc25 was significantly decreased in *ksg1-208*, Δ*ppk21*, or Δ*cdr2* cells, and overexpression of Ppk21 or Cdr2 partially recovered the decreased protein level of Cdc25 in the *ksg1-208* mutant. Altogether, our findings indicated that Cdr2 is a novel downstream effector of PDK1 homologs Ksg1 and Ppk21, both of which cooperatively participate in regulating cell cycle progression, and Cdc25 is involved in this process in fission yeast.

## Introduction

The eukaryotic cell cycle, as one of the most important and evolutionary conserved processes of cells, plays a crucial role in the growth, development, repair, and reproduction of living organisms. Dysregulation of cell cycle procession caused by mutation or aberrant gene expression can cause a myriad of cellular pathologies including defects in cell shape, abnormal cell growth and aneuploidy, potentially leading to the occurrence and development of cancer (Abbastabar et al., 2018; Zheng et al., 2019). Therefore, a better understanding of regulatory mechanisms on cell cycle procession will provide a theoretical basis for further research on cell cycle-related diseases and promote the development of therapeutic strategies.

The 3-phosphoinositide-dependent protein kinase 1 (PDK1) is a widely conserved serine/threonine-protein kinase in eukaryotes. Alterations of PDK1 functions have a relevant role in pathologies such as Alzheimer’s disease, diabetes, and cancer (Hashimoto et al., 2006; Pietri et al., 2013; Jiang Q. et al., 2021). Increasing pieces of evidence have shown that the expression of PDK1 is dysregulated in multiple cancer types, and PDK1 is implicated in signaling pathways frequently altered in cancer (Gagliardi et al., 2012; Zabkiewicz et al., 2014; Bai et al., 2016; Wang et al., 2016). PDK1 has been known to play a central role in cellular signaling by phosphorylating members of the AGC family of kinases, including protein kinase C (PKC), protein kinase B (PKB), p70/p90 ribosomal S6 kinases (RSK and S6K), and the catalytic subunit of cAMP-dependent protein kinase (PKA) (Alessi et al., 1997; Stokoe et al., 1997; Mora et al., 2004). Several other proteins not belonging to the AGC kinase family were also reported to participate in the PDK1 signaling pathway (Gagliardi et al., 2015), however, the detailed mechanisms are not well understood. Therefore, further studies are still required to gain an insight into the PDK1 signaling pathway, which may pave a way for further understanding the molecular mechanisms of the PDK1 regulatory network and providing clues for rational PDK1 targeting therapies in the future.

The fission yeast *Schizosaccharomyces pombe* (*S. pombe*) has been known for years to be an excellent model system for the investigation of the fundamental regulations and control mechanisms of various cellular processes, due to its high homology with mammals and advantages in gene manipulation (Forsburg, 2005; Liu et al., 2018, 2020; Jiang G. et al., 2021; Vyas et al., 2021). Ksg1 (kinase responsible for sporulation and growth 1), which is essential for the cell viability in fission yeast, is a homologous protein of mammalian PDK1 (Niederberger and Schweingruber, 1999). It has been reported that decrease of Ksg1 kinase activity reduces cell wall integrity and mating efficiency of fission yeast significantly, which is mediated by the Ksg1 substrates Pck1/Pck2 (pombe C kinase-like 1/2) and Pka1 (protein kinase A1) (Matsuo et al., 2003; Tang and Mcleod, 2004; Madrid et al., 2014). Remarkably, the Ksg1 substrates in fission yeast are homologous to that of PDK1 in mammalian cells (Kobayashi and Cohen, 1999; Gagliardi et al., 2018), suggesting the significant conservation between Ksg1 and PDK1 signaling pathways. Thus, further studies of the Ksg1-regulated pathway could provide novel references for the PDK1 signaling network in higher eukaryotes. A previous study showed that a mutation of Ksg1 leads to the cell cycle arrest in G2/M transition under high temperature (Niederberger and Schweingruber, 1999), indicating the crucial role of Ksg1 in cell cycle regulation. However, the underlying mechanism by which Ksg1 regulates cell cycle progression has not been reported so far.

In this study, in an attempt to excavate new elements and reveal novel signaling pathways for Ksg1 in regulating cell cycle, we screened for multicopy suppressors of *ksg1-208* mutant cells. Two genes encoding serine/threonine-protein kinases were identified, namely the SAD (synapses of the amphid defective) kinase Cdr2 (changed division response 2) (Young and Fantes, 1987; Crump et al., 2001) and another PDK1 homolog Ppk21 (putative protein kinases 21, also known as Pdk1) (Bimbó et al., 2005a). We investigated the role of Ppk21 and Cdr2 in the Ksg1-mediated cell cycle progression, and found that Ksg1 and Ppk21 cooperatively regulate cell cycle progression through Cdr2. Moreover, overexpression of *ppk21*^+^ or *cdr2*^+^ restored the decreased protein level of a mitotic inducer Cdc25 in the *ksg1-208* mutant. Our results revealed a novel function of Ppk21 and Cdr2 in Ksg1-regulated cell cycle progression, which provides a theoretical basis for enriching the PDK1-associated regulatory network in humans. To our knowledge, this is the first demonstration of a cross-talk between PDK1 signaling and SAD kinase signaling.

## Materials and Methods

### Strains, Media, and Genetic and Molecular Biology Methods

The *S. pombe* strains used in this study are listed in Table 1. The complete medium yeast extract-peptone-dextrose (YPD) and the normal minimal medium Edinburgh minimal medium (EMM) have been described previously (Toda et al., 1997). Standard *S. pombe* genetic and recombinant-DNA methods were performed as described previously unless mentioned (Moreno et al., 1991). Gene disruptions are denoted by lower-case letters representing the disrupted gene followed by two colons and the wild-type gene marker used for disruption (for example, *cdr2*::*ura4*^+^). Gene disruptions are abbreviated by the gene preceded by Δ (for example, Δ*cdr2*). Proteins are denoted by Roman letters and only the first letter is capitalized (for example, Cdr2).

**TABLE 1:** Strains used in this study.

Strain	Genotype	References
HM123	*h*^–^ *leu1-32*	Our stock
KP810	*h*^+^ *leu1-32 ura4-D18 cdc25*^+^-*6HA::ura4^+^::cdc25-22*	Furnari et al., 1997
KP2803	*h*^–^ *leu1-32 ksg1-208*	Our stock
KP3094	*h*^–^ *leu1-32 ura4-D18 ppk21::ura4^+^*	This study
KP3155	*h*^–^ *leu1-32 ura4-D18 ksg1-208 ppk21::ura4^+^*	This study
KP3473	*h*^+^ *leu1-32 his2 ura4-D18 ksg1-208*	Our stock
KP3474	*h*^–^ *leu1-32 ura4-D18 ksg1-208*	Our stock
KP3687	*h*^+^ *leu1-32 his2 ura4-D18 cdr2::ura4^+^*	This study
KP3700	*h*^–^ *leu1-32 ura4-D18 ksg1-208 cdr2::ura4*^+^	This study
KP3769	*h*^–^ *leu1-32 ura4-D18 cdr2::ura4*^+^	This study
KP4746	*h^+^ leu1-32 ade6-M21X ura4-D18 ppk21::KanMX_4_*	Our stock
KP5555	*h*^+^ *leu1-32 ura4-D18 ade6-M21X cdr2-mEGFP::kanMX_6_*	Moseley et al., 2009
KP5558	*h*^–^ *cdr2::natR*	Our stock
KP93006	*h^+^ leu1-32 ade6-M21X ura4-D18*	Kim et al., 2010
CM121	*h*^–^ *leu1-32 cdr2-mEGFP::KanMX_6_*	This study
CM122	*h*^–^ *leu1-32 ksg1-208 cdr2-mEGFP::KanMX_6_*	This study
CM126	*h*^–^ *leu1-32 ura4-D18 ppk21::ura4^+^ cdr2-mEGFP::KanMX_6_*	This study
CM128	*h*^–^ *leu1-32 ura4-D18 ksg1-208 ppk21::ura4^+^ cdr2-mEGFP::KanMX_6_*	This study
CM244	*h*^–^ *leu1-32 ura4-D18 ksg1-208 cdc25*^+^-*6HA::ura4^+^::cdc25-22*	This study
CM568	*h*^–^ *leu1-32 ura4-D18 ppk21::KanMX_4_ cdc25^+^-6HA::ura4^+^::cdc25-22*	This study
CM574	*h*^–^ *leu1-32 ura4-D18 cdr2::natR cdc25^+^-6HA::ura4^+^::cdc25-22*	This study

### Multicopy Suppressor Screen

To identify multicopy suppressor of the thermosensitivity of *ksg1-208* mutant, a genomic library cloned into the vector pDB248 (Beach et al., 1982) was transformed into the *ksg1-208* mutant cells. The Leu^+^ transformants were replica-plated onto YPD plates and incubated at 35°C for 4 days. The plasmid DNA was recovered from transformants that showed a plasmid-dependent rescue. Using DNA sequencing and sequence alignment, the suppressing genes were confirmed. Here we focus on the genes *cdr2*^+^ (SPAC57A10.02) and *ppk21*^+^ (SPBC1778.10c).

### Gene Deletion

To knockout the *cdr2*^+^ gene, a one-step gene disruption by homologous recombination was performed (Rothstein, 1989). The *cdr2*::*ura4*^+^ disruption was constructed as follows. The 2.3-kb *cdr2*^+^ fragment with *Bam*HI site at both ends, which was amplified by PCR using primers *cdr2*^+^ sense/ *cdr2*^+^ antisense (Table 2) and the genomic DNA of wild-type cells as a template, was subcloned into the *Bam*HI site of BlueScriptSK(+). Then, an *ura4*^+^ fragment was inserted into the *Sph*I site of the previous construct. The fragment containing the disrupted *cdr2*^+^ gene was transformed into haploid cells. Stable integrants were selected on medium without uracil, and the disruption was confirmed by PCR and Southern blot (our unpublished data). We generated *ksg1-208* Δ*cdr2* double mutant by a genetic cross between *ksg1-208* and *cdr2*::*ura4*^+^.

**TABLE 2:** Primers used in this study.

Primer name	Sequence (5′–3′)
*cdr2*^+^ sense	CGCGGATCCGATGAGTACAATTTCAGAAGTTGGACCTTGG
*cdr2*^+^ antisense	CGCGGATCCGGTTAACTTTGGACGGATTGTCGTTGACG
*ppk21*^+^ sense	CCGCTCGAGATGGATCTGGAGCATAAACGC
*ppk21*^+^ antisense	CGGGATCCGCGGCCGCCCTCTTCCTCGTTCTCTTCTAC

*Underlined letters represented the restriction sites.*

To knockout the *ppk21*^+^ gene, we used the similar method to that of Cdr2 disruption. The 1.9-kb *Xho*I/*Not*I fragment containing *ppk21*^+^ gene was amplified by PCR using primers *ppk21*^+^ sense/ *ppk21*^+^ antisense (Table 2) and the genomic DNA of wild-type cells as a template, and then was inserted into the *Xho*I/*Not*I sites of BlueScriptSK(+). Then, *BamH*I digested *ura4*^+^ fragment was inserted into the *Bgl*II site of *ppk21*^+^ in the previous construct. The *ura4*^+^ disrupted *ppk21*^+^ fragment was transformed into haploid cells, and the stable integrants were selected on medium without uracil, and the disruption was confirmed by PCR and Southern blot (our unpublished data). A genetic cross was employed between *ksg1-208* and *ppk21*::*ura4*^+^ to generate *ksg1-208* Δ*ppk21* double mutant.

### Gene Expression

To construct overexpression plasmids, the *cdr2*^+^ and *ppk21*^+^ genes were amplified by PCR using the genomic DNA of wild-type cells as a template. The primers were used as mentioned above (Table 2). Then, the *cdr2*^+^ fragment was inserted into the *Bam*HI restriction site of the pREP1 expression vector, which harbors a thiamine-repressible *nmt1*^+^ promoter, while the *ppk21*^+^ fragment was inserted into the *Xho*I and *Not*I restriction sites of the pREP1 expression vector. These constructs were validated by sequencing, and were further confirmed to be fully functional as demonstrated by their complementation of the phenotypes associated with Δ*cdr2* or Δ*ppk21*, respectively (our unpublished data).

To examine the effect of Ksg1 or Ppk21 on the subcellular localization or protein abundance of Cdr2, we constructed the chromosome integrated strains by genetic cross using strain expressing Cdr2-mEGFP with *ksg1-208*, *ppk21*::*ura4*^+^ or their double mutant, respectively, to generate *ksg1-208 cdr2-mEGFP*::*KanMX*_6_, *ppk21*::*ura4^+^ cdr2-mEGFP*::*KanMX*_6_, or *ksg1-208 ppk21::ura4^+^ cdr2-mEGFP::KanMX_6_.*

To investigate the effect of Ksg1, Ppk21, or Cdr2 on the abundance of Cdc25 protein, we constructed CM244, CM568, and CM574 by genetic cross between *cdc25-6HA*::*ura4*^+^ and *ksg1-208*, *ppk21*::*KanMX*_4_ or *cdr2*::*natR*, respectively.

### Microscopy and Miscellaneous Methods

Methods in light microscopy, such as fluorescence microscopy and differential interference contrast (DIC) microscopy, were performed as described previously (Kita et al., 2004; Fang et al., 2008) by using a Nikon Eclipse Ni-U microscope equipped with a DS-Qi2 camera (Nikon Instruments Inc., Japan). Cell length and septation index were measured using the ImageJ software.^1^ Database searches were performed using the National Center for Biotechnology Information BLAST network service and the fission yeast *S. pombe* database search service.^2^

### Immunoblot Assays

Cells expressing Cdr2-mEGFP or Cdc25-HA under wild-type or mutant background were cultured at 27°C in normal EMM media with 0.226 g/L leucine overnight to mid-log phase. The *cdr2*^+^ or *ppk21*^+^ overexpressed Cdc25-HA cells were incubated in normal EMM media to mid-log phase. Total proteins were extracted in buffer (92.5% 2 M NaOH, 7.5% β-mercaptoethanol), and precipitated by adding an equal volume of 50% trichloroacetic acid. After washing with Tris-HCl buffer, the resulting protein extracts were dissolved in sample buffer (62.5 mM Tris-HCl, pH 6.8, 2% SDS, 10% glycerine, 1 mM β-mercaptoethanol). Following resolution by SDS-polyacrylamide gel electrophoresis, proteins were transferred to a polyvinylidene difluoride membrane (Millipore, United States) under 150 mA constant current for 1 h in transfer buffer (12.5 mM Tris-HCl, 192 mM glycine, and 10% methanol; pH 8.3). Purified polyclonal anti-GFP an monoclonal anti-HA (Cell Signaling Technology, 1:10,000) antibodies were used to visualize protein bands.

### Statistical Analysis

Quantitative data are expressed as the mean ± standard deviation of the mean (SD). Statistical analyses were performed using GraphPad Prism 8.3.0 (GraphPad Software, La Jolla, CA). One-way ANOVA and Tukey’s multiple range test were conducted to determine the significance of differences. For all statistical analyses, a single asterisk (*) denotes *p*-values < 0.05, two asterisks (^**^) indicate *p*-values < 0.01, three asterisks (^***^) indicate *p*-values < 0.001, and four asterisks (^****^) indicate *p*-value < 0.0001.

## Results

### Isolation of the *ppk21*^+^ and *cdr2*^+^ Genes as Multicopy Suppressors of *ksg1-208* Mutant

**FIGURE 1:**
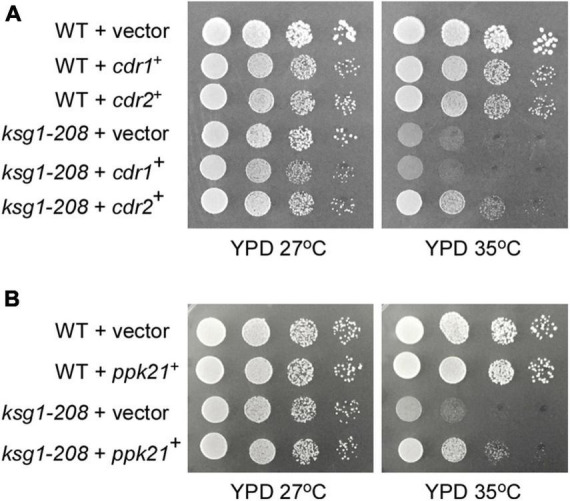
Isolation of Cdr2 and Ppk21 as multicopy suppressors of the *ksg1-208* mutant cells. The wild type cells (WT) or *ksg1-208* mutant cells were transformed with either the pDB248 empty vector or the vector containing *cdr1*^+^, *cdr2*^+^ **(A)** or *ppk21*^+^ **(B)**. Cells were spotted onto YPD plated as indicated in serial 10-fold dilutions starting with OD_660_ = 0.3 of log-phase cells and then incubated for 4 days at 27°C or 35°C.

Ksg1, a homolog of PDK1 which was recognized as a phosphoinositide signaling regulator, was reported to be responsible for the control of cell cycle previously, however, the mechanism remains elusive (Niederberger and Schweingruber, 1999). To identify novel genes that are involved in Ksg1 function, we performed a multicopy suppressor screen of the thermosensitive *ksg1-208* strain. The strain has a single mutation (G159E) between the ATP binding domain and the active site of Ksg1, which results in decreased kinase activity and growth defects at restrictive temperature (Niederberger and Schweingruber, 1999). A high-copy genomic library bearing the *Leu1*^+^ marker (Beach et al., 1982) was used for selecting genes that when overexpressed could rescue the *ksg1-208* growth defects at a restrictive temperature of 35°C. DNA sequencing identified the genomic insert of each suppressing plasmid, and two of them were investigated in this study, namely *ppk21*^+^ and *cdr2*^+^. The *ppk21*^+^ gene encodes a non-essential Pkh (PKB-activating kinase homologs) kinase Ppk21 which is another homologous protein of PDK1 in fission yeast, and the *cdr2*^+^ gene encodes an 85.97 kDa SAD family kinase Cdr2 (Breeding et al., 1998; Kanoh and Russell, 1998; Casamayor et al., 1999; Bimbó et al., 2005b). As shown in Figure 1, the *ksg1-208* cells grew well in the YPD medium at a permissive temperature of 27°C, but failed to grow at 35°C, whereas wild-type cells grew well. Notably, overexpression of either *ppk21*^+^ or *cdr2*^+^ partially rescued the temperature sensitivity of the *ksg1-208* cells at 35°C (Figures 1A,B). We also examined the effects of overexpression of another SAD kinase Cdr1 (Wu and Russell, 1993; Kanoh and Russell, 1998) on the growth defect of *ksg1-208* cells at 35°C, and the results showed that overexpression of *cdr1*^+^ could not rescue the temperature sensitivity of *ksg1-208* cells (Figure 1A). These results indicated that Ppk21 and Cdr2 might be involved in Ksg1-related cell growth regulation.

### Ppk21 Shares a Redundant Function With Ksg1 in Cell Cycle Regulation

**FIGURE 2:**
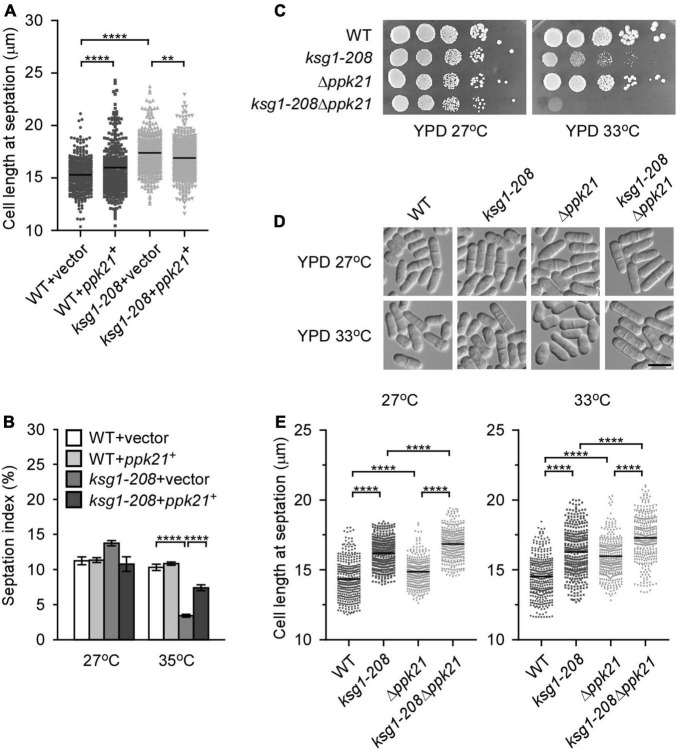
Ppk21 shares a redundant function with Ksg1 in cell cycle regulation. **(A)** Quantification of the cell length at septation for each *ppk21*^+^ overexpressed strains cultured with EMM plated without thiamine. Black bars represent mean (*n* ≥ 300). ** indicates *p*-value < 0.01 and **** indicates *p*-value < 0.0001. **(B)** Septation index of *ppk21*^+^ overexpressed strains cultured with EMM plated without thiamine (*n* ≥ 1000). Data represents mean (± SD) of three biological replicates. **** indicates *p*-value < 0.0001. **(C)** Wild-type, *ksg1-208*, Δ*ppk21* or *ksg1-208* Δ*ppk21* cells were spotted onto YPD plates and then incubated for 4 days at 27°C or 33°C. **(D)** Live-cell images of wild-type, *ksg1-208*, Δ*ppk21*, or *ksg1-208* Δ*ppk21*cells. Cells were grown to early log-phase in YPD plates at 27°C and then transferred to 33°C for 3 h and images were acquired using differential interference contrast (DIC) microscope immediately. Scale bar, 10 μm. **(E)** Quantification of the cell length at septation for each strain as indicated. Black bars represent mean (*n* ≥ 300). **** indicates *p*-value < 0.0001.

A previous study reported that cells deleted for *ppk21*^+^ displayed multiple defects in mitosis (Bimbó et al., 2005b), indicating an important role of Ppk21 in cell cycle regulation. This prompted us to investigate whether overexpression of *ppk21*^+^ played a role in repairing the defective G2/M transition of the *ksg1-208* cells. Fission yeast cells enter into mitosis at a reproducible mean size of 13∼15 μm, and a longer cell length at division indicates the delay of G2/M transition, and vice versa (Mitchison and Nurse, 1985; Scotchman et al., 2021). Then we measured the cell length of the *ksg1-208* cells at division, and determined the effects of Ppk21 overexpression on the cell length of *ksg1-208* cells. Consistent with the notion that Ksg1 is important for cell cycle G2/M transition (Niederberger and Schweingruber, 1999; Matsuo et al., 2003), our results showed that the mean length of *ksg1-208* cells was significantly longer than that of wild-type cells at 27°C (Figure 2A). Remarkably, overexpression of *ppk21*^+^ recovered the defects in the cell length of the *ksg1-208* cells (Figure 2A). In view of the fact that septation initiates after mitosis, and to some extent, the ratio of septation reflects the proportion of cells that have gone through G2/M transition and mitosis, we further determined the effects of Ppk21 overexpression on the septation index of *ksg1-208* cells. As shown in Figure 2B, the septation index of *ksg1-208* cells decreased sharply at 35°C which was recovered by the overexpression of *ppk21*^+^ (Figure 2B). These results indicated that overexpressed *ppk21*^+^ helped the *ksg1-208* cells to pass through the G2/M transition of the cell cycle.

Since Ppk21 is another homologous protein of PDK1 in fission yeast (Bimbó et al., 2005b), we then asked whether Ppk21 functions redundantly with Ksg1 in cell cycle regulation. To determine the role of *ppk21*^+^ in Ksg1-regulated cell cycle progression, we constructed a double mutant of *ksg1*^+^ and *ppk21*^+^, namely *ksg1-208* Δ*ppk21*. We then compared the phenotypes of *ksg1-208* Δ*ppk21* cells with Δ*ppk21* or *ksg1-208* cells. As shown in Figure 2C, the *ksg1-208* Δ*ppk21* cells showed more marked temperature sensitivity than either *ksg1-208* cells or Δ*ppk21* cells (Figure 2C). In addition, we performed the cell length assay, and the results showed that *ksg1-208* Δ*ppk21* cells exhibited a longer cell length than either *ksg1-208* or Δ*ppk21* cells at both 27 and 33°C (Figures 2D,E). These results indicated that deleting the *ppk21*^+^ gene exacerbated the cell cycle delay and temperature sensitivity of the *ksg1-208* mutant, and Ppk21 shares a redundant function with Ksg1 in the cell cycle regulation. Meanwhile, we noticed that the septation ring at 33°C seems off-centered for the *ksg1-208* Δ*ppk21* mutant. To determine whether *ksg1-208* mutation or Ppk21 deletion cause division site mis-positioning, we measured septum position in *ksg1-208*, Δ*ppk21*, and *ksg1-208* Δ*ppk21* cells by quantification of the ratio of the short to long daughter cell as described previously (Willet et al., 2019). The results showed that the septation ring of the *ksg1-208* Δ*ppk21* double mutant was off-centered at 33°C, which was more severe than that of *ksg1-208* cells (Figure 2D and Supplementary Figure 1). Thus, not only G2 arrest but also mis-septation may be responsible for the growth defect of *ksg1-208* Δ*ppk21* double mutant.

### Overexpression of *cdr2*^+^ Recovers the Defect of Cell Cycle Transition in *ksg1-208* Mutant

**FIGURE 3:**
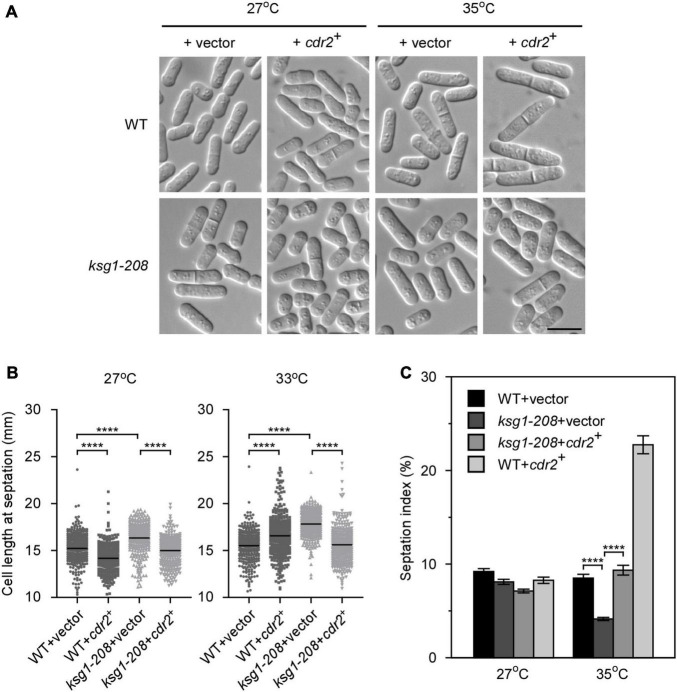
The overexpression of *cdr2*^+^ partially rescues the phenotypes of *ksg1*^+^ mutant cells. **(A)** Live-cell images of wild-type or *ksg1-208* cells overexpressing *cdr2*^+^ gene. Wild-type or *ksg1-208* cells were transformed with pREP1 empty vector or pREP1 vector containing *cdr2*^+^ and grown to early log-phase in EMM plates without thiamine at 27°C. Then cells were transferred to 35°C for 3 h and images were acquired using differential interference contrast (DIC) microscope immediately. Scale bar, 10 μm. **(B)** Quantification of the cell length at septation for each indicated strain grown at 27°C or transferred to 33°C for 3 h. Black bars represent mean (*n* ≥ 300). **** indicates *p*-value < 0.0001. **(C)** Septation index of each indicated strain grown at 27°C or transferred to 35°C for 3 h (*n* ≥ 1000). Data represent mean (± SD) of three biological replicates. **** indicates *p*-value < 0.0001.

The SAD kinase Cdr2 is a key component of the signaling network that prevents mitotic entry until cells reach a critical and reproducible size (Martin and Berthelot-Grosjean, 2009; Moseley et al., 2009; Allard et al., 2018). The pivotal role of Cdr2 in cell cycle regulation prompted us to investigate whether overexpression of *cdr2*^+^ rescued the defect of G2/M transition in *ksg1-208* cells. We found that, similar to the *ppk21*^+^, overexpression of *cdr2*^+^ also reversed the defects in the cell length and the septation index of *ksg1-208* cells (Figures 3A,B), suggesting that the mitotic entry returned to normal in *cdr2*^+^ overexpressed *ksg1-208* cells. It should be noted that *cdr2*^+^ overexpressed wild-type cells showed a shorter cell length at 27°C, but a longer cell length at 35°C than *cdr2*^+^ non-overexpressed wild-type cells (Figure 3B). In addition, the *cdr2*^+^ overexpressed wild-type cells showed a higher septation index than *cdr2*^+^ non-overexpressed wild-type cells at 35°C (Figure 3C). A previous study reported that overexpression of *cdr2*^+^ in the wild-type strain was lethal and generated elongated, highly branched cells that contained two or more septa (Breeding et al., 1998). The differences between the previous report and our present data may be due to the use of wild-type cells with different auxotrophic properties and genetic backgrounds. To this end, we overexpressed *cdr2*^+^ in another wild type strain, KP93006, which possesses the same auxotrophic properties with the strain used in previous report (Kim et al., 2010), and then cultured the cells in the same condition together with the *cdr2*^+^ overexpressed wild type cells HM123 described in Figure 3C. Consistent with the previous report, our results showed that overexpression of *cdr2*^+^ in KP93006 generated elongated and branched cells, whereas overexpression of *cdr2*^+^ in HM123 showed a higher septation index (Supplementary Figure 2). Collectively, our results suggested that overexpression of *cdr2*^+^ rescued the defective cell cycle transition of *ksg1-208* mutant cells.

### Cdr2 Works Downstream of Ksg1 and Ppk21 in the Regulation of Cell Cycle

**FIGURE 4:**
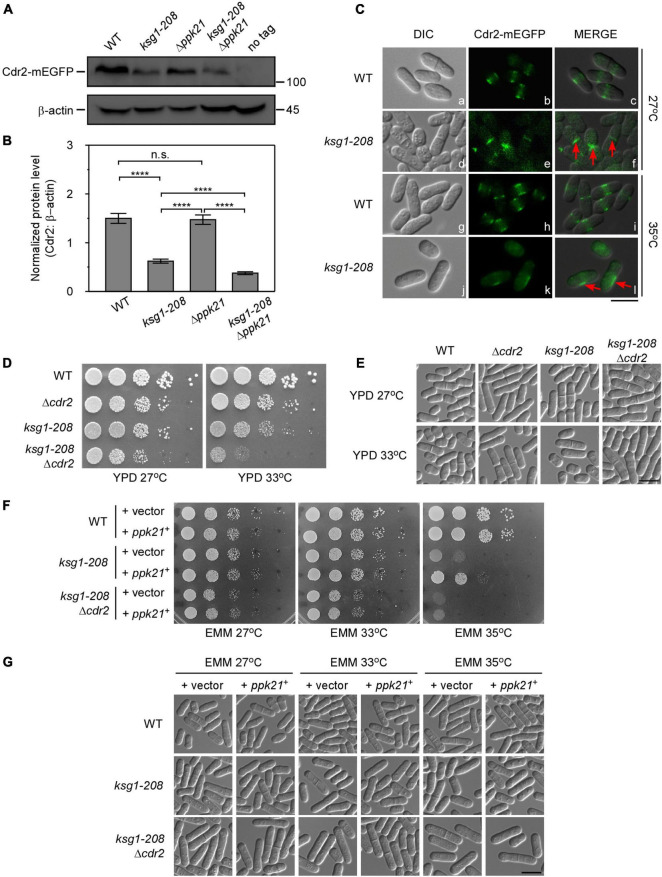
Cdr2 works downstream of Ksg1 and Ppk21. **(A)** Western blots of Cdr2 protein level in wild-type, *ksg1-208*, Δ*ppk21*, and *ksg1-208* Δ*ppk21* cells. Indicated strains were grown to early log-phase in EMM solution with 0.226 g/L leucine but without thiamine at 27°C. A wild-type sample (no tag) was loaded as a control. Total Cdr2 level was blotted by anti-GFP antibody, and β-actin was used as a loading control. **(B)** Quantification of total Cdr2 level in **(A)**. Values were normalized by β-actin, and data represents mean (±SD) of three biological replicates. **** indicates *p*-value < 0.0001 while n.s. indicates no significant differences according to Tukey’s test. **(C)** Subcellular localization of Cdr2 in wild-type or *ksg1-208* mutant cells grown at 27°C or 35°C. Indicated cells were grown to early log-phase in EMM containing 4 μM thiamine at 27°C and then transferred to 35°C for 3 h. Red arrows imply mis-localized Cdr2-mEGFP. Scale bar, 10 μm. **(D)** Wild-type, *ksg1-208*, Δ*cdr2* or *ksg1-208* Δ*cdr2* cells were spotted onto YPD plates and then incubated for 4 days at 27°C or 33°C. **(E)** Live-cell images of strains in **(D)**. Cells were grown to early log-phase in YPD plates and then were transferred to 33°C for 3 h and images were acquired using differential interference contrast (DIC) microscope immediately. Scale bar, 10 μm. **(F)** Overexpressed *ppk21*^+^ failed to rescue the temperature sensitivity of *ksg1-208* mutant when the *cdr2*^+^ gene was deleted. Wild-type, *ksg1-208* or *ksg1-208* Δ*cdr2* cells overexpressing *ppk21*^+^ were spotted onto EMM plates without thiamine and then incubated for 4 days at 27°C, 33°C, or 35°C. **(G)** Live-cell images of strains in **(F)**. Each strain were cultured in EMM plates without thiamine to early log-phase at 27°C and then were transferred to 33°C or 35°C for 3 h, and DIC images were acquired by microscope immediately. Scale bar, 10 μm.

The fact that overexpressed *cdr2*^+^ facilitated the mitotic entry of *ksg1-208* mutant cells prompted us to test the effects of *ksg1*^+^ mutation on the protein level of Cdr2. Using integrated GFP tags, we found that the protein level of Cdr2 in *ksg1-208* cells was significantly lower than that in wild-type cells (Figures 4A,B), indicating that *ksg1-208* mutation reduced the protein abundance of Cdr2. Notably, the protein level of Cdr2 in *ksg1-208* Δ*ppk21* cells was significantly lower than that in *ksg1-208* cells, despite the Cdr2 protein level in Δ*ppk21* cells showed no obvious difference compared with that in wild-type cells (Figures 4A,B). These results suggested that Ksg1 and Ppk21 regulated the abundance of Cdr2 synergistically.

The proper localization is crucial for Cdr2 to control cell cycle progression. Reportedly, Cdr2 forms a central broad band with other cortical node proteins through much of the G2 phase and during very early mitosis (Akamatsu et al., 2014). Then in G1 and S phase, Cdr2 proteins dissociate from the cortex and diffuse in the cytoplasm, and have no specific cell surface localization between septum formation and cell separation (Akamatsu et al., 2014; Rincon et al., 2017). As expected, in wild-type cells, Cdr2-mEGFP localized to the central cortex in the cell middle, whereas diffused to the cytoplasm in the dividing cells (Figure 4C). In contrast, a fraction of *ksg1-208* cells showed septum or division site localized Cdr2-mEGFP in the dividing cells at 27°C, indicating the cortex dissociation of Cdr2 was hindered. Notably, asymmetrical off-centered localization of Cdr2 appeared in the majority of the *ksg1-208* cells at 35°C, and the fluorescence intensity became much weaker than that of the wild-type cells (Figure 4C). These results suggest that Ksg1 acts upstream of Cdr2.

To further explore the relationship between Cdr2 and Ksg1, we constructed the *ksg1-208* Δ*cdr2* cells. As shown in Figure 4D, the *ksg1-208* Δ*cdr2* cells showed more marked temperature sensitivity than that of *ksg1-208* cells or Δ*cdr2* cells. Consistent with this, the cell length of *ksg1-208* Δ*cdr2* cells is longer than that of *ksg1-208* cells (Figure 4E). Importantly, we found that overexpressed *ppk21*^+^ failed to rescue the temperature sensitivity of the *ksg1-208* cells in the absence of the *cdr2*^+^ gene (Figures 4F,G). These results further demonstrate that Ppk21 also acts upstream of Cdr2.

### Overexpression of *ppk21*^+^ or *cdr2*^+^ Partially Reversed the Decreased Cdc25 Level Caused by *ksg1*^+^ Mutation

**FIGURE 5:**
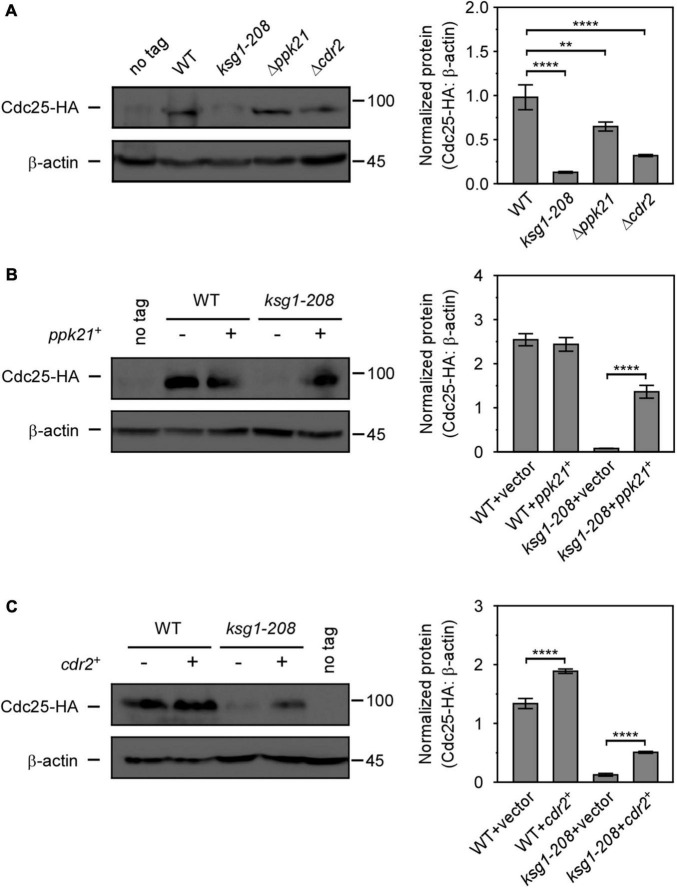
Overexpressed *ppk21*^+^ or *cdr2*^+^ gene partially reversed the decreased Cdc25 level caused by *ksg1*^+^ mutation. **(A)** Western blots (left) and quantification (right) of Cdc25-HA in wild-type, *ksg1-208*,Δ*ppk21* and Δ*cdr2* cells. Cells were grown to early log-phase in EMM solution containing 0.226 g/L leucine but without thiamine at 27°C and then harvested for total protein extraction. **(B,C)** Western blots of Cdc25-HA protein level in *ksg1-208* cells overexpressing *ppk21*^+^ or *cdr2*^+^. A wild-type sample (no tag) was loaded as a control. Total Cdc25 level was blotted by anti-HA antibody, and β-actin was used as a loading control. Quantification of Cdc25 level was normalized by β-actin. Data represents mean (±SD) of three biological replicates. ** indicates *p*-value < 0.01 and **** indicates *p*-value < 0.0001.

Cdc25, a well-known G2/M inducer, was reported to regulate the cell size by size-dependent expression, which provided a mechanism for cells to trigger cell division when they reach a threshold concentration of Cdc25 (Keifenheim et al., 2017). Since *ksg1* mutation led to a longer cell length and a defective G2/M transition, we then sought to investigate whether Cdc25 was involved in Ksg1-regulated cell cycle progression. Western blot analysis showed that the level of Cdc25 protein was dramatically lower in *ksg1-208* cells than that in wild-type cells, indicating that Ksg1 played a crucial role in the accumulation of Cdc25 protein (Figure 5A). In addition, the Cdc25 protein level decreased in Δ*ppk21* and Δ*cdr2* cells as well, indicating the role of Ppk21 and Cdr2 on regulating Cdc25 protein level (Figure 5A). Intriguingly, overexpression of *ppk21*^+^ or *cdr2*^+^ regained the Cdc25 protein level partially in *ksg1-208* cells (Figures 5B,C). Given the regulatory role of Ksg1 and Ppk21 on Cdr2, we concluded that Cdr2 works downstream of Ksg1 and Ppk21 to adjust the Cdc25 protein level.

## Discussion

The PDK1 homolog Ksg1 was identified as an essential kinase in fission yeast responsible for growth, mating, and sporulation by phosphorylating the activation loop of AGC kinases (Niederberger and Schweingruber, 1999; Matsuo et al., 2003; Tang and Mcleod, 2004). It was reported that mutation of *ksg1* leads to growth arrest in the G2/M phase of the cell cycle (Niederberger and Schweingruber, 1999), however, the mechanisms remain unclear. In this study, we identified SAD kinase Cdr2 and another PDK1 homolog Ppk21 as novel elements involved in Ksg1 function, and revealed the important role of these two elements in the process of cell cycle regulated by Ksg1. To our knowledge, this is the first report of the involvement of Ppk21 and Cdr2 in Ksg1-regulated cell cycle progression.

Ppk21 was originally recognized as a key regulator involved in mitosis and cytokinesis (Bimbó et al., 2005b). In this study, several lines of evidence support the idea that Ksg1 and Ppk21 function redundantly in cell cycle regulation. First, similar to Ksg1, Ppk21 is another PDK1 homolog. Second, *ksg1-208* cells exhibited temperature sensitivity, longer cell length, and lower septation index, which were rescued by overexpression of *ppk21*^+^. Third, the *ksg1-208* Δ*ppk21* cells exhibited more marked temperature sensitivity than the *ksg1-208* mutant, although the Δ*ppk21* cells did not show temperature sensitivity. In addition, the *ksg1-208* Δ*ppk21* cells displayed a longer cell length than each single mutant, suggesting that deletion of Ppk21 exacerbated the defective cell cycle transition of *ksg1-208* mutant. All these suggest that Ksg1 and Ppk21 were functionally redundant, and Ksg1 may be the major one in cell cycle regulation. Moreover, *ksg1-208* Δ*ppk21* cells showed enhanced division plane-positioning defects compared to *ksg1-208* cells at 33°C, further indicating the synergistic effect of Ksg1 and Ppk21. It is possible that Ksg1 and Ppk21 share some substrates, thereby leading to the redundant function.

The integration of cell growth and cell cycle progression ensures the reproducible cell division size. The key component of this integration in fission yeast is Cdr2, which organizes cortical nodes in the cell middle to sense cell size and promote mitotic entry (Breeding et al., 1998; Kanoh and Russell, 1998; Martin and Berthelot-Grosjean, 2009; Moseley et al., 2009; Rincon et al., 2017; Allard et al., 2018; Facchetti et al., 2019). The activity of Cdr2 increases as cells grow in the G2 phase while the total level keeps constant throughout the cell cycle (Deng et al., 2014). In addition, the periodic change of Cdr2 localization regulated by phosphorylation status is also a crucial premise for its function (Rincon et al., 2017; Willet et al., 2019). In our study, we found that the protein level of Cdr2 significantly decreased in *ksg1-208* mutant cells (Figures 4A,B), suggesting the important role of Ksg1 in stabilizing the protein level of Cdr2. Besides, anomalies in Cdr2 localization appeared in *ksg1-208* mutant cells at both permissive and restrictive temperatures (Figure 4C). The obvious septum or division site localization of Cdr2 in *ksg1-208* cells at 27°C implied the defects in dissociation from the cortex, similar to the localization of Cdr2 mutated at two SIN-dependent phosphorylation sites (Rincon et al., 2017). The asymmetrical off-centered localization of Cdr2 at 35°C further revealed the defects in the spatial distribution of Cdr2 in *ksg1-208* mutant cells. In particular, the absence of Ppk21 exacerbated the decrease of Cdr2 protein level in *ksg1-208* cells. More importantly, overexpression of *ppk21*^+^ could rescue the temperature sensitivity of *ksg1-208* cells, but failed to rescue the temperature sensitivity when the *cdr2*^+^ gene was deleted. Taken into the effect of overexpressed *cdr2*^+^ on cell length and septation of *ksg1-208* cells, these data together demonstrated that Cdr2 is a novel downstream effector of Ksg1 and Ppk21 in cell cycle control. A previous study reported that overexpression of *cdr2*^+^ was toxic and generated cells that contained two or more septa (Breeding et al., 1998). Consistently, we found that overexpression of *cdr2*^+^ caused the wild-type cells a longer cell length and a higher septation index (Figures 3B,C). Interestingly, overexpression of *cdr2*^+^ did not cause the *ksg1-208* cells a longer length and a higher septation index (Figures 3B,C), suggesting the necessity of Ksg1 activity for the function of Cdr2. This further demonstrated that Cdr2 functions downstream of Ksg1.

Then, how does Ksg1-Ppk21-Cdr2 signaling promote the cell cycle progression? In eukaryotes, the cell cycle G2/M phase transition is controlled by mitotic cyclin-dependent kinase complex (Cdc2-cyclin B), which is inactivated by Wee1 family protein kinases and activated by the opposing phosphatase Cdc25 (Russell and Nurse, 1986, 1987; Karlsson-Rosenthal and Millar, 2006; Glover, 2012). Previous studies have shown that Cdr2 recruits Wee1 to cortical nodes through interacting with the N-terminal of Wee1 and negatively regulates the latter together with another SAD family kinase Cdr1 to promote mitotic entry (Allard et al., 2018). However, a recent study showed that both Cdr1 and Cdr2 promote the phosphorylation of Wee1 *in vivo*, but only Cdr1 inhibits the kinase activity of Wee1 (Opalko et al., 2019). Taken together with our results that overexpression of *cdr2*^+^, but not *cdr1*^+^, rescued the growth defect of *ksg1-208* cells, we speculate that Ksg1 regulates cell cycle progression via Cdr2, which may be independent of modifying the kinase activity of Wee1. Thus, Cdc25 came into our consideration. Cdc25 is a mitotic inducer whose expression is dependent on cell size. The concentration of Cdc25 increases as cells grow and triggers cell division when it reaches a threshold (Keifenheim et al., 2017). In this study, we found that the protein level of either Cdr2 or Cdc25 was significantly lower in *ksg1-208* cells than that in the wild-type cells, indicating that the abundance of both Cdr2 and Cdc25 was repressed when Ksg1 kinase activity was damaged. In addition, the absence of Ppk21 or Cdr2 also reduced the protein level of Cdc25. In particular, overexpression of the *ppk21*^+^ or *cdr2*^+^ gene partially recovered the reduced Cdc25 protein level in *ksg1-208* cells. These results indicated a regulatory role of Ksg1, Ppk21, and Cdr2 on Cdc25, and Ksg1-Ppk21-Cdr2 signaling may promote the cell cycle progression through Cdc25. A previous study reported that loss of Cdr2 activity delays mitosis in strains that have no active Cdc25 protein, and concluded that Cdc25 may not be the primary target of Cdr2 regulation (Kanoh and Russell, 1998). Probably, Cdc25 is regulated slightly but significantly by Cdr2, whereas unidentified factors exist in Ksg1-regulated signaling network to mainly control the Cdc25 abundance. Meanwhile, we could not exclude the possibility that Cdc25 is regulated by Cdr2 indirectly, and unidentified molecular(s) may be involved in this process. The detailed mechanisms need to be further illustrated.

In conclusion, we propose a mechanism by which Ksg1 controls cell cycle progression according to our results: PDK1 homologs Ksg1 and Ppk21 function redundantly and they act upstream of Cdr2, thereby controlling cell cycle progression and Cdc25 is involved in this process in fission yeast. Our findings provide a novel theoretical foundation for enriching the PDK1 regulatory network in humans. Whether Ksg1 and Ppk21 involve in Cdr2 phosphorylation as well as how they regulate Cdc25 are important questions to be addressed in the future.

## Data Availability Statement

The original contributions presented in the study are included in the article/Supplementary Material, further inquiries can be directed to the corresponding author/s.

## Author Contributions

YF designed the study. KL, QL, YS, JF, and YZ performed the experiments. NS and TK analyzed the data. KL and YF wrote the manuscript with suggestions from all authors.

## Conflict of Interest

The authors declare that the research was conducted in the absence of any commercial or financial relationships that could be construed as a potential conflict of interest.

## Publisher’s Note

All claims expressed in this article are solely those of the authors and do not necessarily represent those of their affiliated organizations, or those of the publisher, the editors and the reviewers. Any product that may be evaluated in this article, or claim that may be made by its manufacturer, is not guaranteed or endorsed by the publisher.

## Footnotes

1
https://imagej.nih.gov/ij/

2
http://www.pombase.org/
