# Supplementary material for: Phosphoinositide-Dependent Protein Kinases Regulate Cell Cycle Progression Through the SAD Kinase Cdr2 in Fission Yeast
Source: Front Microbiol. 2022 Jan 10;12:807148. doi: 10.3389/fmicb.2021.807148 (PMC8784684; doi:10.3389/fmicb.2021.807148)
Supplement: Supplementary file 1 [file Data_Sheet_1.docx]

Supplementary Material


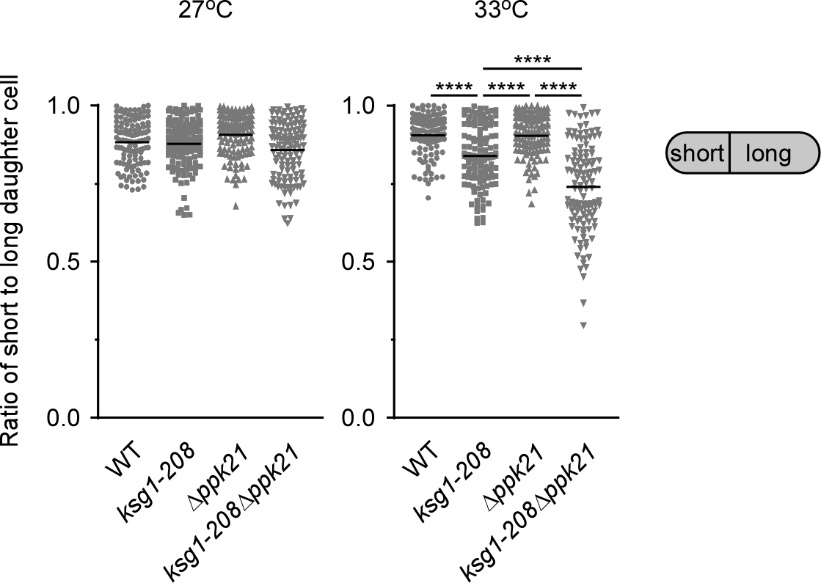


**Supplementary Figure 1.** Quantification of the ratio of the short to long daughter cell for each indicated strain. Cells were cultured in YPD plates to early log-phase at 27^o^C, and then the cells were transferred to 33^o^C for 3 hours. Live-cell images were acquired by differential interference contrast (DIC) microscope immediately. The lengths of short or long daughter cells were measured using the ImageJ software (<https://imagej.nih.gov/ij/>). Black bars represent mean. n ≥100. **** indicates *p* value <0.0001, one-way ANOVA.

**
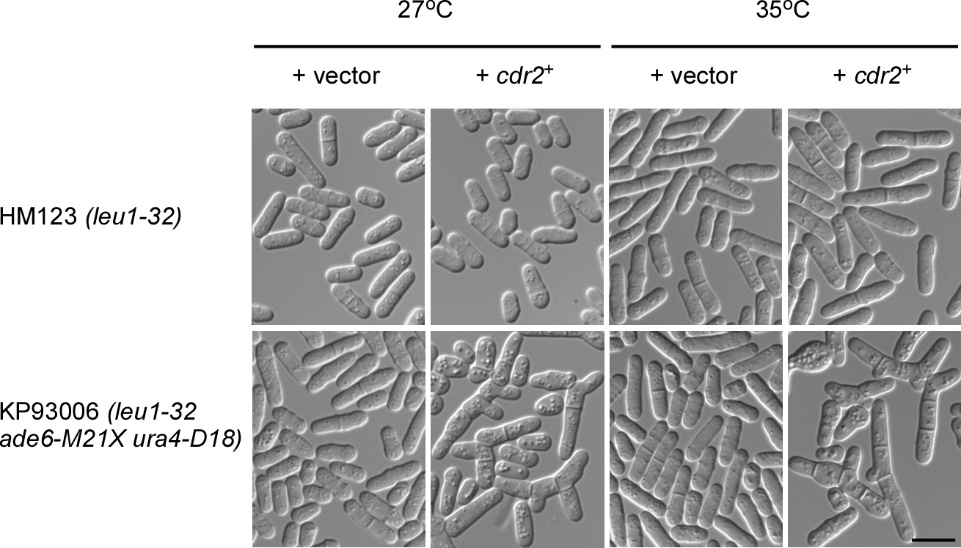
**

**Supplementary Figure 2.** Live-cell images of *cdr2*^+^ overexpressed wild type cells with different auxotrophic properties. HM123 (*leu1-32*) and KP93006 (*leu1-32 ade6-M21X ura4-D18*) were transformed with pREP1 empty vector or pREP1 vector containing *cdr2*^+^ and grown to early log-phase in EMM plates containing adenine and uracil without thiamine at 27^o^C. Then cells were transferred to 35^o^C for 3 hours and images were acquired using differential interference contrast (DIC) microscope immediately. Scale bar, 10 μm.
